# Effectiveness of community mobilisation and group-based interventions for preventing intimate partner violence against women in low- and middle-income countries: A systematic review and meta-analysis

**DOI:** 10.7189/jogh.13.04115

**Published:** 2023-10-20

**Authors:** Jessica Leight, Claire Cullen, Meghna Ranganathan, Alexa Yakubovich

**Affiliations:** 1 Poverty, Gender and Inclusion, International Food Policy Research Institute, Washington DC, USA; 2 Blavatnik School of Government, Oxford University, Oxford, England, UK; 3 Department of Global Health and Development, London School of Hygiene and Tropical Medicine, London, England, UK; 4 Dalhousie University, Department of Community Health and Epidemiology, Faculty of Medicine, Halifax, Nova Scotia, Canada

## Abstract

**Background:**

Intimate partner violence (IPV) is a challenge affecting one in three women in their lifetime, and gender-transformative interventions have been identified as a promising prevention strategy. We systematically reviewed and meta-analysed randomised controlled trials (RCTs) of community-level or group-based interventions to prevent IPV in lower- and middle-income countries, seeking to answer the following research question: do community- or group-based gender-transformative interventions reduce IPV, compared to a control arm of status-quo programming?

**Methods:**

We conducted a systematic search from the inception of all databases employed until 20 July 2021. Eligible study outcomes included past-year experience of physical, sexual, emotional or economic IPV self-reported by women and perpetration of physical or sexual IPV self-reported by men. We assessed study risk of bias using the updated Cochrane tool for RCTs. We estimated the pooled odds ratio (OR) using a multilevel random-effects meta-analysis and also conducted a multilevel meta-regression to analyse how study characteristics moderated the effect size.

**Results:**

After screening 7363 unique records, we included 30 studies on 27 unique RCTs. Our meta-analysis suggested that community-level or group-based interventions reduced the odds of women experiencing IPV in the past year: pooled adjusted odds ratio (aOR) = 0.78; 95% confidence interval (CI) = 0.63-0.97. While there was significant heterogeneity in the effect sizes between trials (*I*^2^ = 83%), potentially reflecting the diverse contexts of the included trials, our meta-regression did not indicate a significant association between intervention effectiveness and intervention type or target population. There was evidence of significant associations between effectiveness and intervention components and duration.

**Discussion:**

There is strong evidence that community-level and group-based interventions reduce IPV against women. Unpacking what intervention modalities are effective in which contexts can further inform prevention strategies.

**Registration:**

PROSPERO (CRD42021290193).

Intimate partner violence (IPV) remains a global human rights and public health challenge. Recent estimates suggest that 27% of ever-partnered women and girls aged 15-49 have ever experienced physical or sexual violence by an intimate partner, with the highest prevalence estimates in low- and middle-income countries (LMICs) [1]. IPV has major implications for the health and well-being of women, their families and communities. Women who have experienced IPV are more likely to report injuries [2], depression and anxiety, and substance abuse [3], and are exposed to higher human immunodeficiency virus (HIV) risk [4].

Even prior to the onset of the coronavirus disease 2019 (COVID-19) pandemic, the world was not on track to meet Sustainable Development Goal (SDG) 3.2 calling for the elimination of IPV, and evidence suggests the COVID-19 pandemic has only increased its prevalence [5]. Conceptual models highlight that violence is the result of the interplay of multiple factors at individual, relationship, community, and societal levels [6]. The unequal position of women in relationships, unequal social norms, and household-level conflict are all risk factors for IPV [7].

Building on these insights, an increasingly large number of gender-transformative interventions using community or group-based strategies targeting the broader drivers of IPV have been developed and evaluated. The definition of gender-transformative intervention employed here follows that employed by the World Health Organization (WHO): a gender-transformative approach “seeks to challenge gender inequality by transforming harmful gender norms, roles and relations through programmatic inclusion of strategies to foster progressive changes in power relationships between women and men” [8,9]. The theory of change for these interventions centres around the premise that shifting community attitudes, social norms and individual behaviours through peer-led or group-based participatory processes is essential for preventing IPV.

Our aim is to conduct a systematic review and meta-analysis drawing on randomised controlled trials (RCTs) of community-level or group-based interventions targeting the prevention and reduction of IPV in LMICs, unified by these shared elements in the theory of change. Previous systematic reviews have included evidence from both high-income and low-income countries [10]; focused on primarily economic interventions [11-14] or interventions that incorporate both economic and social components [15-17]; or only included evidence from Sub-Saharan Africa [18]. The majority of these systematic reviews also included quasi-experimental or non-experimental designs. However, as policymakers are increasingly turning to scalable gender-transformative interventions to tackle IPV prevention, there has been a rapid increase in RCTs evaluating such interventions, and this has generated a gap in meta-analytic evidence.

This review thus seeks to extend and update the evidence base by providing the first comprehensive analysis of high-quality experimental evidence documenting the effectiveness of community and group-based interventions targeting IPV in LMICs. Our research question can be formulated as follows: do community- or group-based gender-transformative interventions targeting the prevention or reduction of women’s experiences of IPV lead to lower rates of IPV, compared to a control arm of status-quo programming?

## METHODS

### Search strategy and selection criteria

We draw on the PRISMA guidelines to report on this systematic review and meta-analysis and have pre-registered our protocol with PROSPERO (CRD42021290193). We searched the following databases: Medline, Web of Science, PsychINFO, EconLit, Scopus, Science Direct, SSRN Research Papers, Econ Papers, Global Health and Social Sciences Abstracts for published papers; the full search strategy is provided in Table S1 in the **Online Supplementary Document**. We used a combination of terms to capture IPV; intervention or programme; communication, prevention, or policy; RCT; and a filter for LMICs. We developed our search strategy based on previous reviews and followed best practice in those reviews in implementing the search to identify relevant terms in the title or abstract as well as using MeSH terms [6,13,18]. We conducted additional searches for grey literature in the World Bank Open Knowledge Repository, the WHO Institutional Repository for Information Sharing and the WHO Violence database. Study searches continued until 20 July 2021.

We defined the inclusion criteria as follows: RCTs described in English and conducted in LMICs, as defined by the World Bank [19], that evaluated community- or group-based gender-transformative interventions targeting the prevention or reduction of women’s experiences of IPV. Exclusion criteria were: trials in high-income countries; interventions targeting female sex workers or adolescents under the age of 18; interventions that were economic only; therapeutic interventions delivered one-on-one to couples or individuals; studies of violence perpetrated against men, violence perpetrated by women or violence within same-gender couples; and studies that exclusively sampled women who had previously experienced IPV. JL screened all abstracts and full-text articles. A second reviewer (CC or MR) double-screened a random 10% and differences were resolved via discussion.

We defined the exclusion criteria in order to focus effectively on interventions that share common elements in the theory of change. The criteria excluded interventions targeting adolescents under 18 given that these interventions often emphasise distinct theories of change centred around building human and social assets [20]. We similarly excluded interventions that are solely economic – for example, cash or asset transfers that primarily target poverty reduction –given that the theory of change for effects on intimate partner violence for these interventions generally emphasises an increase in economic welfare or a reduction in conflict, rather than a shift in underlying gender norms [12]. Interventions that target solely women who report prior experience of IPV, or perpetration of violence by women or within same-gender couples, were similarly excluded given that these samples have distinct relational dynamics.

### Data analysis

We conducted the meta-analysis based on study reports and did not draw on individual participant data. We extracted data using a customised form, including study design (e.g. statistical power/number of clusters); descriptive data on the intervention; setting (country, region, urban/rural); primary outcome; intervention sample size (or multiple sample sizes, if multiple intervention arms); control arm sample size; estimated odds ratio (OR) or risk ratio (RR) for outcomes of interest; estimated confidence intervals; and loss to follow-up. We assessed studies’ risk of bias at the study level using the updated Cochrane risk-of-bias tool for both individual and cluster-randomised trials, and aggregated information using the Excel templates and tools [21]. JL conducted data extraction and risk of bias assessments and CC independently checked the data and assessments for 50% of the included studies.

The outcomes of interest included past-year experience of physical IPV, sexual IPV, emotional IPV, economic IPV, or any IPV, all self-reported by women and measured using standard WHO instruments; and past-year perpetration of physical IPV or sexual IPV, as reported by men. If results were reported for multiple follow-up periods, the estimated treatment effects for the longest follow-up period were extracted. When relevant data were not available in published study reports, JL contacted study authors up to two times to request missing information.

The primary effect measure for this analysis was the OR for an intent-to-treat analysis, coding all individuals randomly assigned to an offer of treatment as treated. For eligible studies that did not report ORs, we used available data to calculate the ratio. In addition, if estimated treatment effects did not appropriately account for clustering, we adjusted for clustering in line with Cochrane guidelines.

We estimated a multi-level (three-level) random-effects meta-analysis model, given that the assumptions for a fixed-level meta-analysis (that all studies shared a common effect size and factors influencing this size are consistent across studies) did not plausibly hold in this context [22,23]. We conducted all computation on a log-scale, before exponentiating the summary effect for interpretation, and used a restricted maximum likelihood method [24]. The multilevel model included all available estimated effects for each trial (both primary and secondary outcomes, reported across multiple arms if applicable) and accounted for the dependence across estimated effects within the same trial. We conducted separate meta-analyses for unadjusted and adjusted estimates according to best practice [25]. In addition, we assessed between-study heterogeneity using the multilevel τ^2^ and *I*^2^ statistics.

We conducted five sensitivity analyses. First, we re-estimated the multi-level model including only treatment estimates for women’s reported experience of violence, excluding treatment estimates for male perpetration. Second, we re-estimated the multi-level model allowing every paper reporting analysis of a separate intervention or sample (even for the same overarching trial) to enter the meta-analysis separately. Third, we estimated a simpler random-effects meta-analysis model using only one estimated coefficient for a primary outcome of interest from each trial. Fourth, we re-estimated the main multi-level model excluding studies characterised by some risk of bias in more than one domain. Fifth, we estimated separate models for each of the six IPV outcomes.

We then estimated meta-regressions using the primary multilevel model in order to estimate the extent to which study characteristics (intervention type, target population, intervention components, and intervention duration) moderate the effect size. We employed separate models for characteristics linked to intervention type and target population and characteristics linked to intervention components and duration. Note that a model including the full set of characteristics is not estimated in order to avoid potential multicollinearity. Further methodological details around the multilevel model and the meta-regression are provided in the **Online Supplementary Document**.

The analysis codes intervention type as community mobilisation; group-based interventions including women; group-based interventions including men; or group-based interventions including couples. Joint interventions constituted the reference group. We define a community mobilisation intervention as an intervention that seeks to provide communication and education across a cross-section of individuals and stakeholders in a given community. We define a group-level intervention as an intervention delivering a pre-established curriculum to defined groups of men, women or couples; in these interventions, individuals who are not part of the defined groups would not be directly targeted. A joint intervention included more than one of these elements: for example, we categorised an intervention that established a group-based intervention for men as well as a parallel but separate group-based intervention for women as a joint intervention.

The analysis coded the target population as youth (defined as individuals aged 18 to 30 years), cohabiting couples, or the reference group of an unrestricted target population. While interventions targeting only adolescents under 18 years were not eligible for this review based on the inclusion criteria, interventions targeting youth over 18 were eligible and the review identified a number of trials that specifically targeted youth characterised by a maximum age of 25 or 30.

The analysis also coded intervention components to identify additional thematic foci of the intervention, including HIV and sexual and reproductive health (SRH), substance use, economic empowerment, and parenting. A focus on HIV/SRH indicates the intervention also provided information or behavioral change counseling around topics related to HIV/SRH; a focus on substance use indicates the intervention also targeted the use of alcohol or other substances and the interrelationships between substance use and IPV; a focus on economic empowerment indicates the intervention also included a dimension seeking to build livelihoods or enhance economic welfare; and a focus on parenting indicates the intervention also targeted enhanced parenting practices or the reduction of violence against children. The reference group is interventions that do not include any of these additional components.

Finally, we coded intervention duration using two methods: the number of contact hours for participants (if reported) and the total duration in years of intervention activities (if reported). Both variables were dichotomised to generate binary variables defined as high intensity (above the median contact hours observed across interventions) and long duration (above the median duration observed across interventions). We used the meta and metafor packages of R version 4.1.3 for analysis [26].

## RESULTS

**Figure 1:**
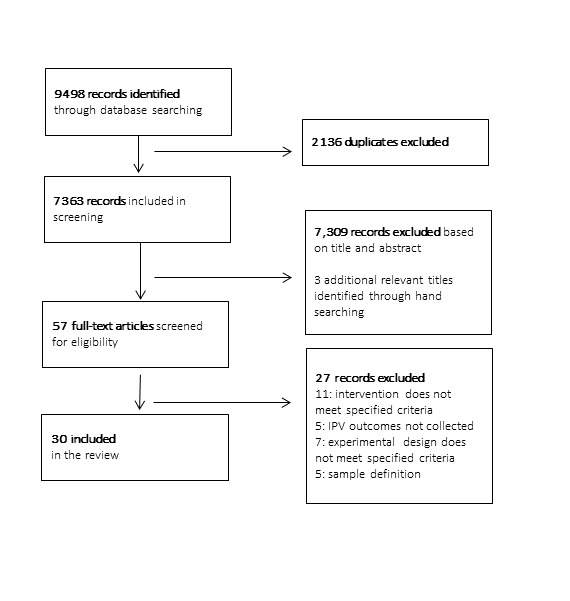
PRISMA flow diagram.

The literature search identified 7363 unique records. After title and abstract screening, we screened the full texts of 57 potentially relevant studies and identified 30 as eligible for inclusion in the review (**Figure 1**). We included 29 studies in the meta-analysis, but excluded one study due to the absence of key details required to interpret the statistical findings [27]. **Table 1** summarises the included studies’ key characteristics. Most studies were conducted in Sub-Saharan Africa (n = 26, 87%) [27-52], with three studies (10%) from Asia [53-55] and one study (3%) from Latin America [56]. Studies from South Africa (n = 8, 27%) [32,35,38,44,45,47,48,51] were most common.

**Table 1:** Characteristics of included studies

Study	Country	Intervention	Num. of treatment arms	Sampling frame	Sample size for analysis	Outcomes measured	Method of clustering
Abramsky et al. (2014)	Uganda	SASA!	1	Men/women of reproductive age	8 clusters, 2532 individuals	Experience: physical IPV, sexual IPV	Site-level analysis conducted
Abramsky et al. (2016)	Uganda	SASA!	1	Men/women of reproductive age	8 clusters, 2532 individuals	Experience of emotional IPV	Site-level analysis conducted
Chatterji et al. (2020)	Rwanda	Indashyikirwa	1	Men/women of reproductive age who are partnered	28 clusters, 2800 individuals	Experience: physical, sexual, emotional, economic IPV. Perpetration: physical, sexual IPV	Unit of randomization included as a random effect
Christofides et al. (2020)	South Africa	Sonke CHANGE	1	Men of reproductive age	18 clusters, 1508 individuals	Perpetration: physical, sexual IPV	Cluster-level analysis performed
Clark et al. (2020)	Nepal	Change Starts at Home	1	Married women of reproductive age	36 clusters, 1436 individuals	Experience: physical, sexual, emotional, economic IPV	Degrees of freedom account for nesting
Doyle et al. (2018)	Rwanda	Bandebereho	1	Men aged 21-25 who are married, cohabiting, or fathers, and their partners	2285 individuals	Experience: physical, sexual IPV	Robust standard errors with clustering by facilitator
Dunkle et al. (2020)	Rwanda	Indashyikirwa	1	Women and their male partners of reproductive age who are partnered at least 6 mo and active VSLA members	28 clusters, 3153 individuals	Experience: physical, sexual, emotional, economic IPV. Perpetration: physical, sexual IPV.	Unit of random-isation included as a random effect
Fawzi et al. (2019)	Tanzania	Namweza	1	Men and women who are above age 18, HIV+ and willing to serve as change agents, and individuals in their social networks who are not already receiving HIV care	NR	Experience: physical, sexual IPV. Perpetration: physical, sexual IPV	NA
Ferrari et al. (2010)	Burundi	VSLA-based course on household decision-making	1	Women of reproductive age who are VSLA members	NR	Experience of physical IPV	Standard errors clustered at the village level
Gibbs et al. (2020a)	South Africa	Stepping Stones and Creating Futures	1	Men and women between age 18—30 who are not in school, education or formal employment	34 clusters, 1050 individuals	Experience: physical, sexual, emotional, economic IPV. Perpetration: physical, sexual IPV	Not specified, but states accounted for clustering
Gibbs et al. (2020b)	Afghan-istan	Woman for Women International 12-mo economic and social empowerment programme	1	Women of reproductive age who are in households earning less than $1.25 a day and who are not economically active or engaged in education	1210 individuals	Experience: physical, emotional IPV	NA
Gupta et al. (2013)	Cote d'Ivoire	Gender Dialogue Groups	1	Women of reproductive age	24 clusters, 934 individuals	Experience: physical, sexual, economic IPV	Cluster included as random effect
Halim et al. (2019)	Tanzania	Male peer groups	2	Women of reproductive age who are partnered and savings group members, and their male partners	9 clusters, 740 individuals	Experience: physical, sexual, emotional, economic IPV. Perpetration: physical, sexual IPV	Robust standard errors clustered by village
Harvey et al. (2021)	Tanzania	Maisha	1	Women of reproductive age	66 clusters, 1126 individuals	Experience: physical, sexual, emotional IPV	Cluster included as random effect
Hossain et al. (2014)	Cote d'Ivoire	Men's Discussion Groups	1	Men 15 y or older, and their female partners	12 clusters, 560 individuals	Experience: physical, sexual IPV	Site-level analysis conducted
Jewkes et al. (2008)	South Africa	Stepping Stones	1	Men and women aged 15-26	70 clusters, 2221 individuals	Experience: any physical and/or sexual IPV. Perpetration: any physical and/or sexual IPV	Cluster included as random effect
Jones et al. (2014)	South Africa	PartnerPlus	1	Pregnant women (24-30 weeks gestation, at least 18 y of age) and their male partners	12 clusters, 478 individuals	Experience: physical IPV	NA
Kapiga et al. (2021)	Tanzania	Maisha	1	Women of reproductive age who are members of a microfinance group in which at least 70% of all members agreed to participate	66 clusters, 919 individuals	Experience: physical, sexual, emotional IPV	Cluster included as random effect
Maman et al. (2020)	Tanzania	Business training and loan, and peer leader training around GBV/IPV	1	Men of reproductive age who are members of camps in Dar-es-Salaam who meet specified criteria	60 clusters, 1029 individuals	Perpetration: physical IPV	General estimating equations were used to account for clustering
Minnis et al. (2015)	South Africa	Health Co-Op (Men's Health Coop and Women's Health Coop in one arm; Couples’ Health Coop in one arm)	2	Black men aged 18-35 reporting regular alcohol use and unprotected sex, and partnered at least a year	40 clusters, 255 individuals	Experience: any IPV (physical, sexual or emotional)	NA
Naved et al. (2018)	Bangladesh	SAFE	2	Women aged 15-29	Number of clusters NR, 2670 individuals	Experience: physical, sexual, emotional, economic IPV	Random effect for cluster included
Ogum et al. (2020)	Ghana	Rapid Response System	1	Men/women of reproductive age	4 clusters, 4526 individuals	Experience: physical, sexual IPV. Perpetration: physical, sexual IPV	Cluster-level analysis performed
Pettifor et al. (2018)	South Africa	Gender-transformative community mobilisation	1	Men/women of reproductive age	22 clusters, 1175 individuals	Experience of any IPV (physical, sexual or emotional)	Robust variance matrix accounting for clustering
Pronyk et al. (2006)	South Africa	IMAGE	1	Women of reproductive age	8 clusters, 538 individuals	Experience of any IPV (physical, sexual or emotional)	Not specified
Settergren et al. (2018)	Tanzania	WRP/HJFMRI gender-based violence intervention	1	Women of reproductive age	12 clusters, 1143 individuals	Experience: physical, sexual, emotional, any IPV	Cluster included as random effect
Sharma et al. (2020)	Ethiopia	Unite for a Better Life	3	Women of reproductive age who are partnered, and their husbands	64 clusters, 10 379 individuals	Experience: physical, sexual, emotional IPV. Perpetration: physical, sexual IPV	Standard errors clustered at the village level
Skar et al. (2021)	Colombia	Child development programme supplemented by violence prevention curriculum	2	Female caregivers of children aged 3-4, and belonging to specified social service centres	176 individuals	Experience of any IPV (physical, sexual or emotional)	NA
Vaillant et al. (2020)	Congo DRC	EMAP	1	Men of reproductive age, and their partners	28 clusters, 2378 individuals	Experience: any IPV, sexual, emotional, economic IPV	Standard errors clustered
Wagman et al. (2015)	Uganda	SHARE	1	Men/women of reproductive age	11 clusters, 6526 individuals	Experience: physical, sexual, emotional IPV. Perpetration: physical, sexual IPV.	Random effect for cluster included
Wechsberg et al. (2013)	South Africa	Women's Co-op	2	Women aged 18-33 who are regular drug users	604 individuals	Experience: physical IPV	NA

IPV – intimate partner violence, GBV – gender-based violence, VSLA – village savings and loans association, NR – not reported, NA – not applicable

Within these 30 included studies, two papers published primary and secondary outcomes from the same trial (SASA!) [28,29]; another two papers published estimated effects from different samples participating in an interrelated intervention (Indashyikirwa) [31,34]; and a final two papers published estimated effects for two separate cohorts that were also part of the same intervention (Maisha) [39,40] with some minor differences in the implementation strategy. In the latter two cases, the individuals sampled were non-overlapping (in the example of SASA!, the two papers report on the same sample). Given that these correspond to evaluations of the same intervention, we analysed each pair of papers as part of the same trial. A subsequent sensitivity analysis will re-estimate the primary meta-analysis allowing the two studies from the Indashyikirwa and Maisha trials, respectively, to enter the sample as separate trials.

We thus categorised the primary sample as consisting of 27 unique trials, of which three included two intervention arms [37,44,53] and one included three intervention arms [42]. Two additional trials included a second intervention arm that had no explicit focus on prevention or reduction of intimate partner violence or transformation of gender norms and we excluded these additional arms based on the review’s selection criteria [51,56]. The different arms of the 27 trials analysed a total of 32 separate interventions. These included: five community-level mobilisation interventions [28,32,46,47,50], four couples’ interventions [27,33,42,44], five men’s group-level interventions [37,41-43,52], six women’s group-level interventions [30,39,40,42,48,51,56], and 12 joint interventions [31,34-38,44,45,49,53-55].

The interventions target diverse samples: there were seven trials that enrolled samples of cohabiting couples only [31,33,34,37,42,44,45,54] and five studies that enrolled samples of youth only (defined as no more than 30 years old) [33,35,38,44,53]. Two enrolled samples of cohabiting couples among youth [33,44]. There were 15 interventions that included an additional thematic focus on HIV/SRH [28,29,32,33,35-38,42-45,47,48,50,51]; seven that included an additional focus on substance use [31,32,34,35,44,47,51,54]; four that included an additional focus on economic empowerment [35,43,48,55]; and three that included an additional focus on parenting [33,37,56]. In terms of duration, 24 trials reported information around the intervention’s contact hours, and 27 reported information about the intervention’s full period of implementation in years. The median reported contact hours were 36 (minimum four, maximum 104) and the median reported intervention duration was 0.77 years (minimum 0.02 years or one week, maximum four years). Details of intervention coding are summarised in Table S2 in the **Online Supplementary Document**.

**Table 2** summarises the risk of bias for the included studies. In general, we assessed the risk of bias to be relatively low given that the inclusion criteria for the systematic review entailed restricting to RCTs, and more specifically to RCTs that also met other relevant methodological criteria (i.e. the use of appropriate instruments to measure IPV and report associated treatment effects). The main bias-related concerns for the studies we identified were allocation concealment, blinding of enumerators, and the absence of a published protocol or registered pre-analysis plan. As with many community interventions, blinding of participants, implementers, and data collectors either was infeasible or was not addressed [13]. In addition, some studies did not disclose whether the allocation sequence was concealed until participants were enrolled and assigned to the intervention or control group. Overall, consistent with other published meta-analyses of IPV prevention interventions, we find most included RCTs were characterised by either low risk of bias or some risk of bias (most commonly in the domain of selection of the reported result, reflecting the absence of a pre-analysis plan) [13,17,18,57]. No included study was identified as characterised by a high level of bias. A subsequent sensitivity analysis will re-estimate the primary meta-analysis excluding any studies that are characterised by some risk of bias in two or more domains.

**Table 2:** Risk of bias in the included studies

Study	D1a: randomisation process	D1b: timing of identification or recruitment of participants	D2: deviations from the intended interventions	D3: missing outcome data	D4: measurement of the outcome	D5: Selection of the reported result	Overall
Abramsky et al. (2014)	Low*	Low	Low	Low	Low	Low	Low
Abramsky et al. (2016)	Low	Low	Low	Low	Low	Low	Low
Chatterji et al. (2020)	Low	Low	Low	Low	Low	Low	Low
Christofides et al. (2020)	Low	Low	Low	Low	Low	Some†	Some
Clark et al. (2020)	Low	Low	Low	Low	Low	Low	Low
Doyle et al. (2018)	Low	Low	Low	Low	Low	Some	Some
Dunkle et al. (2020)	Low	Low	Low	Low	Low	Some	Some
Fawzi et al. (2019)	Low	Low	Low	Low	Low	Some	Some
Ferrari et al. (2010)	Low	Low	Low	Low	Low	Some	Some
Gibbs et al. (2020)	Low	Low	Low	Low	Low	Low	Low
Gibbs et al. (2020)	Low	Low	Low	Low	Low	Low	Low
Gupta et al. (2013)	Low	Low	Low	Low	Low	Some	Some
Halim et al. (2019)	Low	Low	Low	Low	Low	Some	Some
Harvey et al. (2019)	Low	Low	Low	Low	Low	Low	Low
Hossain et al. (2014)	Some	Low	Low	Low	Low	Some	Some
Jewkes et al. (2014)	Low	Low	Low	Low	Low	Low	Low
Jones et al. (2013)	Low	Low	Low	Low	Low	Some	Some
Kapiga et al. (2021)	Low	Low	Low	Low	Low	Low	Low
Maman et al. (2020)	Low	Low	Low	Low	Low	Some	Some
Minnis et al. (2015)	Low	Low	Low	Low	Low	Some	Some
Naved et al. (2018)	Some	Some	Low	Low	Low	Some	Some
Ogum Alangea et al. (2020)	Low	Low	Low	Low	Low	Low	Low
Pettifor et al. (2018)	Low	Low	Low	Low	Low	Low	Low
Pronyk et al. (2006)	Low	Low	Low	Low	Low	Low	Low
Settergren et al. (2018)	Low	Low	Low	Low	Low	Some	Some
Sharma et al. (2020)	Low	Low	Low	Low	Low	Low	Low
Skar et al. (2021)	Some	Some	Some	Low	Low	Some	Some
Vaillant et al. (2020)	Some	Low	Low	Low	Low	Some	Some
Wagman et al. (2015)	Low	Low	Low	Low	Low	Some	Some
Wechsberg et al. (2013)	Low	Low	Low	Low	Low	Some	Some

*“Low” indicates low risk of bias in the denoted domain.

†“Some” indicates some risk of bias in the domain.

### Meta analysis results

**Figure 2:**
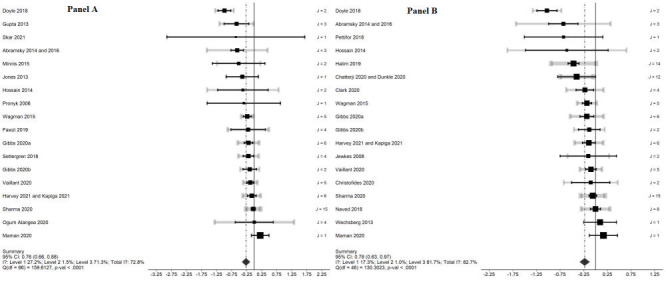
Effect of community- or group-based interventions on intimate partner violence (all estimated effects; unadjusted study-level estimates and adjusted study-level estimates). **Panel A**. Unadjusted study-level estimates. **Panel B**. Adjusted study-level estimates. The plots are generated by estimating a separate meta-analysis for each trial using a random-effects model; the black line for each trial corresponds to the summary outcome for the within-trial meta-analysis. The gray confidence interval captures the median sampling variance observed for the individually estimated effect sizes for a given trial, and the thickness of the confidence interval is increased for trials reporting a large number of outcomes. For trials that include only a single estimated effect, the two confidence intervals are identical. (The parameter J reported on the right indicates the number of effect sizes reported in a given trial.) The weight assigned to each trial in the overall random effects model is captured by the size of the central dot. CI – confidence interval, df – degrees of freedom, *P*-val – *P*-value

From the 29 studies included in the meta-analyses, we summarised 111 unique effect estimates, including 66 unadjusted estimates from 18 trials and 46 adjusted estimates from 18 trials. The multilevel meta-analyses of both the unadjusted and adjusted estimates indicated that community-level or group-based interventions reduced IPV against women in LMICs (unadjusted OR (uOR) = 0.76, 95% CI = 0.66-0.88; adjusted OR (aOR) = 0.78, 95% CI = 0.63-0.97), as reported in **Figure 2**, Panel A for unadjusted estimates and **Figure 2**, Panel B for adjusted estimates [58] The estimated variance components were τ^2^_Level 3_ = 0.07 and τ^2^_Level 2_ = 0.002 in the unadjusted model and τ^2^_Level 3_ = 0.10 and τ^2^_Level 2_ = 0.001 in the adjusted model.

In the analysis of overall variance, sampling error variance comprised 27% of total variance in the unadjusted model and 17% in the adjusted model. Variance within studies across estimated effects was minimal (less than 2% in both models). Between-study heterogeneity thus comprised the majority of the total variation in effect sizes: *I*^2^ = 71% in the unadjusted model and *I*^2^ = 83% in the adjusted model. This suggests a high level of cross-study heterogeneity, using the general guidance of *I*^2^ = 75% as a cutoff for high heterogeneity [59].

In a first sensitivity analysis, we re-estimated the main multilevel model including only treatment effect estimates derived from reports of women’s experience of IPV, excluding treatment effect estimates for male perpetration. Two trials are then dropped from the meta-analysis as they report only treatment effect estimates for perpetration variables [32,43]. These results are reported in Figure S1 in the **Online Supplementary Document** and show very similar findings (uOR = 0.73, 95% CI = 0.63-0.86; aOR = 0.75, 95% CI = 0.61-0.94). All figures for the sensitivity tests can be found in the **Online Supplementary Document**.

In a second sensitivity analysis, we re-estimated the main multilevel model allowing for different papers reporting analysis of related interventions from the same overarching trials to enter the analysis as separate trials. In particular, the two trials analysing different samples as part of Indashyikirwa now enter the meta-analysis separately [31,34], and the two papers analysing two separate cohorts as part of Maisha now enter the meta-analysis separately [39,40]. These results are reported in Figure S2 in the **Online Supplementary Document** and again show consistent findings (uOR = 0.77, 95% CI = 0.67-0.88; aOR = 0.80, 95% CI = 0.66-0.97).

In a third sensitivity analysis, we estimated a simpler random-effects meta-analysis model restricted to include a single coefficient from each trial. In trials that included multiple arms, we selected the arm providing the most intensive intervention. In trials that included multiple outcome variables, we selected a single outcome variable prioritising the outcome variables as prespecified in the protocol: women’s past-year experience of physical IPV, women’s past-year experience of sexual IPV, women’s past-year experience of any IPV (physical, sexual or emotional), women’s past-year experience of economic IPV, men’s past-year perpetration of physical IPV, and men’s past-year perpetration of sexual IPV. The findings are reported in Figure S3 in the **Online Supplementary Document** and show findings that are again consistent with the main multilevel model (uOR = 0.77, 95% CI = 0.64-0.92; aOR = 0.78, 95% CI = 0.63-0.96).

In a fourth sensitivity analysis, we re-estimated the main meta-analysis model excluding four studies identified as characterised by some risk of bias in two or more domains, as summarised in **Table 2** [41,52,53,56]. The findings are reported in Figure S4 in the **Online Supplementary Document** and again show findings consistent with the main model (uOR = 0.75, 95% CI = 0.64-0.89; aOR = 0.78, 95% CI = 0.60-1.00).

In addition, we conducted separate meta-analyses for each outcome variable of interest: women’s past-year experience of physical IPV, women’s past-year experience of sexual IPV, women’s past-year experience of emotional IPV, women’s past-year experience of economic IPV, men’s past-year perpetration of physical IPV, and men’s past-year perpetration of sexual IPV. These findings are reported in Figures S5-S10 in the **Online Supplementary Document**. In general, the findings are consistent across the different outcome variables, though there is variation in sample size and the associated precision of the pooled effect estimate. Focusing on the ORs estimated using adjusted estimates, community-level or group-based interventions reduced experience of physical IPV (aOR = 0.79, 95% CI = 0.65-0.96); experience of sexual IPV (aOR = 0.80, 95% CI = 0.67-0.95); experience of emotional IPV (aOR = 0.81, 95% CI = 0.69-0.95); and perpetration of sexual IPV (aOR = 0.82, 95% CI = 0.68-0.98). The reductions in experience of economic IPV (aOR = 0.75, 95% CI = 0.50-1.14) and perpetration of physical IPV (aOR = 0.91, 95% CI = 0.78-1.05) were similar in magnitude but noisily estimated.

The results from the multilevel meta-regression (**Table 3**) demonstrate that there was no evidence that intervention type was associated with intervention effectiveness. However, studies that evaluated interventions targeting youth compared to those without age- or cohabitation-related sample restrictions showed larger reductions in IPV (OR = 0.62 in model 2 including adjusted effect estimates), though the association was variable (95% CI = 0.35-1.09). There was evidence that some intervention components were associated with effectiveness: interventions including a component targeting parenting practices showed larger reductions in IPV (OR = 0.36 in model 4 including adjusted effect estimates, 95% CI = 0.23-0.57); interventions including a component targeting substance use showed a similar pattern (OR = 0.54 in model 4), though the association was variable (95% CI = 0.27-1.05). Interventions of long duration (above the median of 0.77 years) were also weakly associated with larger reductions in IPV (OR = 0.70 in model 4, 95% CI = 0.48-1.02).

**Table 3:** Meta-regression analysis of intervention effect size*

Variables	Risk ratio (95% CI)	N (trials)	N (estimates)
**Model 1: meta-regression of unadjusted estimates**			
Intervention type (referent: joint intervention)			
*Women*	1.004 (0.660-1.525)	6	16
*Men*	1.093 (0.722-1.655)	5	13
*Couples*	0.973 (0.590-1.606)	4	8
*Community*	0.863 (0.544-1.369)	5	12
Target population (referent: no restrictions)			
*Youth*	0.728 (0.454-1.143)	5	10
*Cohabiting*	0.920 (0.614-1.380)	8	20
Intercept	0.843 (0.628-1.132)		
Test of moderators (coefficients 2:7):			
F (df1 = 6, df2 = 60) = 0.782, *P* = 0.587			
**Model 2: meta-regression of adjusted estimates**			
Intervention type (referent: joint intervention)			
*Women*	0.984 (0.537-1.802)	6	12
*Men*	0.826 (0.454-1.502)	5	20
*Couples*	0.839 (0.456-1.542)	4	7
*Community*	0.623 (0.327-1.188)	5	11
Target population (referent: no restrictions)			
*Youth*	0.616 (0.347-1.094)	5	19
*Cohabiting*	0.832 (0.467-1.483)	8	41
Intercept	1.088 (0.656-1.084)		
Test of moderators (coefficients 2:7):			
F (df1 = 6, df2 = 40) = 2.131, *P* = 0.071			
**Model 3: meta-regression of unadjusted estimates**			
Intervention components (referent: no additional components)			
*HIV/SRH*	1.024 (0.839-1.250)		
*Substance use*	0.736 (0.496-1.092)		
*Economic empowerment*	1.214 (0.902-1.634)		
*Parenting*	0.396 (0.267-0.586)		
Intervention duration (referent: low intensity, short duration)			
*High intensity*	1.276 (1.031-1.579)		
*Long duration*	0.853 (0.683-1.062)		
Intercept	0.732 (0.602-0.890)		
Test of moderators (coefficients 2:7):			
F(df1 = 6, df2 = 60) = 4.851, *P* = 0.0004			
**Model 4: meta-regression of adjusted estimates**			
Intervention components (referent: no additional components)			
*HIV/SRH*	1.134 (0.815-1.580)		
*Substance use*	0.536 (0.274-1.050)		
*Economic empowerment*	1.502 (0.946-2.387)		
*Parenting*	0.363 (0.229-0.574)		
Intervention duration (referent: low intensity, short duration)			
*High intensity*	1.108 (0.801-1.533)		
*Long duration*	0.700 (0.483-1.017)		
Intercept	0.816 (0.619-1.076)		
Test of moderators (coefficients 2:7):			
F (df = 6, df2 = 40) = 4.159, *P* = 0.0024			

CI – confidence interval, df – degrees of freedom, F- F-test - HIV – human immunodeficiency virus, SRH – sexual and reproductive health

*The table reports the results from meta-regressions of unadjusted estimates (models 1, 3) and adjusted estimates (models 2, 4) analysing the moderating effects of the intervention type (women’s group-level intervention, men’s group-level intervention, couples’ group-level intervention, community mobilisation; referent group is a joint intervention); the target population (youth or cohabiting; referent group is unrestricted); the intervention components (additional components linked to HIV/SRH, substance use, economic empowerment, or parenting; referent group is no additional components); and the intervention duration (high intensity, long duration; referent group is low intensity and short duration).

## DISCUSSION

To our knowledge, this is the first systematic, meta-analytic review that provides causal evidence on the effectiveness of gender-transformative IPV prevention programmes in LMICs. The 30 studies included in the review showed that these interventions led to a significant reduction in past-year IPV against women. These interventions are thus potentially effective programmatic tools to prevent and reduce violence experienced by women in a broad range of diverse contexts.

Our review is also the first to employ meta-regression to analyse whether characteristics of the intervention or the target population moderate effectiveness. The findings did not indicate any differences in the effectiveness of different community- or group-based intervention models in reducing IPV, though interventions targeting youth did generate meaningfully larger reductions in IPV, as did interventions incorporating additional thematic foci linked to substance use and parenting. There is also some evidence that programmes characterised by a longer duration generated larger reductions in IPV. Overall, both interventions centred around community mobilisation and those targeting women, men and couples directly seemed equally effective in reducing violence.

While several previous systematic reviews analysed economic interventions such as cash transfers or economic empowerment interventions [12-14], there has been a notable evidence gap around the effectiveness of non-economic interventions, including a lack of meta-analytic evidence [15,18]. Our analysis thus extends the existing literature by aggregating evidence from a clearly defined set of gender-transformative interventions evaluated in RCTs and providing an up-to-date overview of a rapidly growing evidence base. Prior systematic reviews without meta-analyses suggested that there was potential for these strategies to be effective in reducing IPV in LMICs, and our meta-analytic results confirm this finding using more robust methods [15,18].

Our findings further build on two recent systematic reviews and meta-analyses. A previous meta-analysis of RCTs analysing psychosocial interventions for IPV in LMICs found that these interventions reduced any form of IPV by 25% at longest follow-up [57]. However, the definition of the interventions of interest was distinct (encompassing counselling and educational interventions) and the sample significantly smaller (13 trials); the analysis reports on a search conducted in 2017, while in our sample, 20 of the 30 papers included were published in 2018 or later. A second related meta-analysis aggregates effect estimates for any IPV prevention intervention from both experimental and quasi-experimental studies, and found small effects on overall levels of IPV (Cohen’s d = -0.077) [17]. In contrast, our analysis demonstrates that gender-transformative community or group-based interventions that are unified by a defined theory of change and evaluated using the most rigorous designs are in fact effective in preventing and reducing IPV.

From a methodological perspective, an innovative aspect of this review is our use of a multilevel model that pooled all available data from any IPV-related outcome. Our findings indicate that heterogeneity in effect sizes across different IPV outcomes in the same studies was very limited, relative to the variation in estimated effect sizes between studies, suggesting that the analysis of separate forms of IPV may not be necessary in all trials. This is consistent with psychometric evidence suggesting that many IPV scales should be analysed as unidimensional rather than multi-factorial outcomes [60].

Another important implication of our findings emerges from the substantial heterogeneity we observed across studies. This variation was consistent with the level of between-study variance observed in three other recent meta-analyses of IPV prevention interventions, suggesting that context and programmatic factors are likely important in predicting the effects of these interventions [17,57]. More specifically, a meta-analysis of IPV prevention interventions in LMICs reported a *I*^2^ of 77% for IPV behaviour [17]; a meta-analysis of economic empowerment interventions targeting IPV reported values of *I*^2^ generally ranging between 77% and 92% (with only one subanalysis showing a lower *I*^2^ of 55%) for various IPV outcomes and intervention types [13]; and a meta-analysis specifically of psychosocial interventions targeting IPV prevention reported values of *I*^2^ of between 78% and 81% when all RCTs and outcomes were included [57]. Extremely high levels of heterogeneity are also observed in other meta-analyses analysing psychosocial outcomes in LMICs: for example, two recent meta-analyses of mental health interventions reported *I*^2^ of 95% [61] and between 79% and 84% [62]. Accordingly, the high level of heterogeneity reported here is not unique to the particular intervention types or the inclusion and exclusion criteria employed.

Here, we seek to understand further this pattern of high heterogeneity by presenting additional findings – novel in this literature – suggesting that intervention components and duration may moderate these highly variable intervention effects. In light of this evidence, it is particularly important for IPV prevention interventions in the future to include qualitative work that enables researchers to test specific assumptions in the theory of change and understand the elements of the intervention design or context that are resulting in the observed effects.

Our review adds to the existing evidence base of meta-analyses by systematically synthesising evidence from rigorously designed evaluations of community-level and group-based interventions for IPV prevention in LMICs. By using multilevel models in conjunction with meta-regression, we aggregate evidence about both the pooled effect sizes and the relationship between the estimated effect sizes and intervention and population characteristics, a novel contribution in the IPV meta-analysis literature.

### Limitations

Despite its strengths, this study has several limitations. We only searched and included studies available in English. We did not conduct a meta-analysis of intervention effects on non-IPV outcomes such as attitudes or social norms, variables often analysed in these studies; such a meta-analysis may be challenging given that measurement of these outcomes is generally not standardised. In addition, the wide range of diverse contexts in which included trials are conducted may render it more challenging to identify underlying similarities in treatment effects across contexts. The effect estimates from the included trials may also reflect some bias due to selection into the evaluation sample or social desirability bias in reporting, though our risk-of-bias assessment suggests these risks are generally low.

## CONCLUSIONS

This review provides robust causal evidence on the effectiveness of group-based and community-level programmes to prevent IPV. Future research should seek to unpack which elements of these interventions work, for whom, and in what contexts: in particular, mixed-methods research can usefully explore the mechanisms through which community and group-based programmes reduce IPV, as well as monitoring and analysing local trends in IPV prevalence. Experimental designs that include both group-based and community-level programme components may be particularly useful to assess the additive benefits of each type of programme. In addition, we need studies that conduct cost-effectiveness analyses of these programmes to inform policy. The available evidence suggests that both group-based and community-level interventions have promise for reducing IPV in LMICs, and the IPV research agenda should continue to build upon this evidence.

## Additional material


Online Supplementary Document
