# Supplementary material for: Effectiveness of community mobilisation and group-based interventions for preventing intimate partner violence against women in low- and middle-income countries: A systematic review and meta-analysis
Source: J Glob Health. 2023 Oct 20;13:04115. doi: 10.7189/jogh.13.04115 (PMC10588291; doi:10.7189/jogh.13.04115)
Supplement: Online Supplementary Document [file jogh-13-04115-s001.pdf]

Supplementary material

Search strategy

Supplementary Table S1 summarizes the search strategy used in Medline, and adapted for use in other databases. Note the search strategy uses “tw” to identify key words that appear in the title and/or abstract, consistent with the search strategy used in other recent systematic reviews focusing on IPV.[1]

Table S1: Search strategy for Medline

1	violence.tw	
2	abuse*.tw	
3	maltreat*.tw	
4	aggress*.tw	
5	batter*.tw	
6	assault*.tw	
7	beat*.tw	
8	victim.*tw	
9	domestic.tw	
10	partner.tw	
11	partners.tw	
12	marriage*.tw	
13	marital.tw	
14	spouse*.tw	
15	spousal.tw	
16	intimate.tw	
17	husband*.tw	
18	wife.tw	
19	wives.tw	
20	dating.tw	
21	or/1-9	Violence
22	or/10-20	Intimate partner
23	21 and 22	IPV

24	IPV.tw	
25	relationship violence.tw	
26	battered women/	
27	spouse abuse/	
28	exp intimate partner violence/	
29	or/24-28	IPV
30	23 or 29	Final IPV
31	wome?n.tw	
32	girl*.tw	
33	female*.tw	
34	women/	
35	or/31-34	Women
36	intervention*.tw	
37	program*.tw	
38	training.tw	
39	communication.tw	
40	prevent*.tw	
41	policy.tw	
42	policies.tw	
43	or/36-42	Inter-ventions
44	randomized controlled trial [pt]	
45	controlled clinical trial [pt]	
46	randomized [tiab]	
47	placebo [tiab]	
48	drug therapy [sh]	
49	randomly [tiab]	
50	trial [tiab]	
51	groups [tiab]	
52	or/44-51	
53	animals/ NOT humans/	

54	52 NOT 53	RCT
55	afghanistan/ OR albania/ OR algeria/ OR american samoa/ OR angola/ OR antigua and barbuda/ OR argentina/ OR armenia/ OR aruba/ OR azerbaijan/ OR bahrain/ OR bangladesh/ OR barbados/ OR republic of belarus/ OR belize/ OR benin/ OR bhutan/ OR bolivia/ OR bosnia and herzegovina/ OR botswana/ OR brazil/ OR bulgaria/ OR burkina faso/ OR burundi/ OR cabo verde/ OR cambodia/ OR cameroon/ OR central african republic/ OR chad/ OR chile/ OR china/ OR colombia/ OR comoros/ OR democratic republic of the congo/ OR congo/ OR costa rica/ OR cote d'ivoire/ OR croatia/ OR cuba/ OR cyprus/ OR czech republic/ OR djibouti/ OR dominica/ OR dominican republic/ OR ecuador/ OR egypt/ OR el salvador/ OR equatorial guinea/ OR eritrea/ OR estonia/ OR swaziland/ OR ethiopia/ OR fiji/ OR gabon/ OR gambia/ OR georgia (republic)/ OR ghana/ OR gibraltar/ OR greece/ OR grenada/ OR guam/ OR guatemala/ OR guinea/ OR guinea bissau/ OR guyana/ OR haiti/ OR honduras/ OR hungary/ OR india/ OR indonesia/ OR iran/ OR iraq/ OR jamaica/ OR jordan/ OR kazakhstan/ OR kenya/ OR democratic people's republic of korea/ OR republic of korea/ OR kosovo/ OR kyrgyzstan/ OR laos/ OR latvia/ OR lebanon/ OR lesotho/ OR liberia/ OR libya/ OR lithuania/ OR macau/ OR republic of north macedonia/ OR madagascar/ OR malawi/ OR malaysia/ OR indian ocean islands/ OR mali/ OR malta/ OR micronesia/ OR palau/ OR mauritania/ OR mauritius/ OR mexico/ OR moldova/ OR mongolia/ OR montenegro/ OR morocco/ OR mozambique/ OR myanmar/ OR namibia/ OR nepal/ OR netherlands antilles/ OR nicaragua/ OR niger/ OR nigeria/ OR oman/ OR pakistan/ OR panama/ OR papua new guinea/ OR paraguay/ OR peru/ OR philippines/ OR poland/ OR portugal/ OR puerto rico/ OR romania/ OR russia/ OR rwanda/ OR samoa/ OR sao tome and principe/ OR saudi arabia/ OR senegal/ OR serbia/ OR seychelles/ OR sierra leone/ OR slovakia/ OR slovenia/ OR melanesia/ OR somalia/ OR south africa/ OR south sudan/ OR sri lanka/ OR saint kitts and nevis/ OR saint lucia/ OR saint vincent and the grenadines/ OR sudan/ OR suriname/ OR syria/ OR tajikistan/ OR tanzania/ OR thailand/ OR timor leste/ OR togo/ OR tonga/ OR trinidad and tobago/ OR tunisia/ OR turkey/ OR turkmenistan/ OR uganda/ OR ukraine/ OR uruguay/ OR uzbekistan/ OR vanuatu/ OR venezuela/ OR vietnam/ OR middle east/ OR yemen/ OR yugoslavia/ OR zambia/ OR zimbabwe/ OR africa south of the sahara/ OR africa, central/ OR africa, northern/ OR africa, southern/ OR africa, eastern/ OR africa, western/ OR west indies/ OR indian ocean islands/ OR caribbean region/ OR central america/ OR latin america/ OR south america/ OR asia, central/ OR asia, northern/ OR asia, southeastern/ OR asia, western/ OR europe, eastern/ OR developing countries/	
56	afghanistan.tw OR albania.tw OR algeria.tw OR american samoa.tw OR angola.tw OR antigua.tw OR barbuda.tw OR argentina.tw OR armenia.tw OR armenian.tw OR aruba.tw OR azerbaijan.tw OR bahrain.tw OR bangladesh.tw OR barbados.tw OR belarus.tw OR byelarus.tw OR belorussia.tw OR byelorussian.tw OR belize.tw OR british honduras.tw OR benin.tw OR dahomey.tw OR bhutan.tw OR bolivia.tw OR bosnia.tw OR herzegovina.tw OR botswana.tw OR bechuanaland.tw OR brazil.tw OR brasil.tw OR bulgaria.tw OR burkina faso.tw OR burkina fasso.tw OR upper volta.tw OR burundi.tw OR urundi.tw OR cabo verde.tw OR cape verde.tw OR cambodia.tw OR kampuchea.tw OR khmer republic.tw OR cameroon.tw OR cameron.tw OR cameroun.tw OR central african republic.tw OR ubangi shari.tw OR chad.tw OR chile.tw OR china.tw OR colombia.tw OR comoros.tw OR comoro islands.tw OR mayotte.tw OR congo.tw OR zaire.tw OR costa rica.tw OR cote d'ivoire.tw OR cote d' ivoire.tw OR cote divoire.tw OR cote d ivoire.tw OR ivory coast.tw OR croatia.tw OR cuba.tw OR cyprus.tw OR czech republic.tw OR czechoslovakia.tw OR djibouti.tw OR french somaliland.tw OR dominica.tw OR dominican republic.tw OR ecuador.tw OR egypt.tw OR united arab republic.tw OR el salvador.tw OR equatorial guinea.tw OR spanish guinea.tw OR eritrea.tw OR estonia.tw OR eswatini.tw OR swaziland.tw OR ethiopia.tw OR fiji.tw OR gabon.tw OR gabonese republic.tw OR gambia.tw OR georgia.tw OR georgian.tw OR ghana.tw OR gold coast.tw OR gibraltar.tw OR greece.tw OR grenada.tw OR guam.tw OR guatemala.tw OR guinea.tw OR guyana.tw OR guiana.tw OR haiti.tw OR hispaniola.tw OR honduras.tw OR hungary.tw OR india.tw OR indonesia.tw OR timor.tw OR iran.tw OR iraq.tw OR isle of man.tw OR jamaica.tw OR jordan.tw OR kazakhstan.tw OR kazakh.tw OR kenya.tw OR korea.tw OR kosovo.tw OR kyrgyzstan.tw OR kirghizia.tw OR kirgizstan.tw OR kyrgyz republic.tw OR kirghiz.tw OR laos.tw OR lao pdr.tw OR lao people's democratic republic.tw OR latvia.tw OR lebanon.tw OR lesotho.tw OR basutoland.tw OR liberia.tw OR libya.tw OR libyan arab jamahiriya.tw OR lithuania.tw OR macau.tw OR macao.tw OR	

	macedonia.tw OR madagascar.tw OR malagasy republic.tw OR malawi.tw OR nyasaland.tw OR malaysia.tw OR maldives.tw OR indian ocean.tw OR mali.tw OR malta.tw OR micronesia.tw OR kiribati.tw OR marshall islands.tw OR nauru.tw OR northern mariana islands.tw OR palau.tw OR tuvalu.tw OR mauritania.tw OR mauritius.tw OR mexico.tw OR moldova.tw OR moldovian.tw OR mongolia.tw OR montenegro.tw OR morocco.tw OR ifni.tw OR mozambique.tw OR portuguese east africa.tw OR myanmar.tw OR burma.tw OR namibia.tw OR nepal.tw OR netherlands antilles.tw OR nicaragua.tw OR niger.tw OR nigeria.tw OR oman.tw OR muscat.tw OR pakistan.tw OR panama.tw OR papua new guinea.tw OR paraguay.tw OR peru.tw OR philippines.tw OR philipines.tw OR phillipines.tw OR phillippines.tw OR poland.tw OR polish people's republic.tw OR portugal.tw OR portuguese republic.tw OR puerto rico.tw OR romania.tw OR russia.tw OR russian federation.tw OR ussr.tw OR soviet union.tw OR union of soviet socialist republics.tw OR rwanda.tw OR ruanda.tw OR samoa.tw OR pacific islands.tw OR polynesia.tw OR samoan islands.tw OR sao tome and principe.tw OR saudi arabia.tw OR senegal.tw OR serbia.tw OR seychelles.tw OR sierra leone.tw OR slovakia.tw OR slovak republic.tw OR slovenia.tw OR melanesia.tw OR solomon island.tw OR solomon islands.tw OR norfolk island.tw OR somalia.tw OR south africa.tw OR south sudan.tw OR sri lanka.tw OR ceylon.tw OR saint kitts and nevis.tw OR st kitts and nevis.tw OR saint lucia.tw OR st lucia.tw OR saint vincent.tw OR st vincent.tw OR grenadines.tw OR sudan.tw OR suriname.tw OR surinam.tw OR syria.tw OR syrian arab republic.tw OR tajikistan.tw OR tadjikistan.tw OR tadzhikistan.tw OR tadzhik.tw OR tanzania.tw OR tanganyika.tw OR thailand.tw OR siam.tw OR timor leste.tw OR east timor.tw OR togo.tw OR togolese republic.tw OR tonga.tw OR trinidad.tw OR tobago.tw OR tunisia.tw OR turkey.tw OR turkmenistan.tw OR turkmen.tw OR uganda.tw OR ukraine.tw OR uruguay.tw OR uzbekistan.tw OR uzbek.tw OR vanuatu.tw OR new hebrides.tw OR venezuela.tw OR vietnam.tw OR viet nam.tw OR middle east.tw OR west bank.tw OR gaza.tw OR palestine.tw OR yemen.tw OR yugoslavia.tw OR zambia.tw OR zimbabwe.tw OR northern rhodesia.tw OR global south.tw OR africa south of the sahara.tw OR sub saharan africa.tw OR subsaharan africa.tw OR central africa.tw OR north africa.tw OR northern africa.tw OR magreb.tw OR maghrib.tw OR sahara.tw OR southern africa.tw OR east africa.tw OR eastern africa.tw OR west africa.tw OR western africa.tw OR west indies.tw OR indian ocean islands.tw OR caribbean.tw OR central america.tw OR latin america.tw OR south america.tw OR central asia.tw OR north asia.tw OR northern asia.tw OR southeastern asia.tw OR south eastern asia.tw OR southeast asia.tw OR south east asia.tw OR western asia.tw OR east europe.tw OR eastern europe.tw OR developing country.tw OR developing countries.tw OR developing nation.tw OR developing nations.tw OR developing population.tw OR developing populations.tw OR developing world.tw OR less developed country.tw OR less developed countries.tw OR less developed nation.tw OR less developed nations.tw OR less developed world.tw OR lesser developed countries.tw OR lesser developed nations.tw OR under developed country.tw OR under developed countries.tw OR under developed nations.tw OR under developed world.tw OR underdeveloped country.tw OR underdeveloped countries.tw OR underdeveloped nation.tw OR underdeveloped nations.tw OR underdeveloped population.tw OR underdeveloped populations.tw OR underdeveloped world.tw OR middle income country.tw OR middle income countries.tw OR middle income nation.tw OR middle income nations.tw OR middle income population.tw OR middle income populations.tw OR low income country.tw OR low income countries.tw OR low income nation.tw OR low income nations.tw OR low income population.tw OR low income populations.tw OR lower income country.tw OR lower income countries.tw OR lower income nations.tw OR lower income population.tw OR lower income populations.tw OR underserved countries.tw OR underserved nations.tw OR underserved population.tw OR underserved populations.tw OR under served population.tw OR under served populations.tw OR deprived countries.tw OR deprived population.tw OR deprived populations.tw OR poor country.tw OR poor countries.tw OR poor nation.tw OR poor nations.tw OR poor population.tw OR poor populations.tw OR poor world.tw OR poorer countries.tw OR poorer nations.tw OR poorer population.tw OR poorer populations.tw OR developing economy.tw OR developing economies.tw OR less developed economy.tw OR less developed economies.tw OR underdeveloped economies.tw OR middle income economy.tw OR middle income economies.tw OR low income economy.tw OR low income economies.tw OR lower income economies.tw OR low gdp.tw OR low gnp.tw OR low gross domestic.tw OR low gross national.tw OR lower gdp.tw OR lower gross domestic.tw OR lmic.tw OR	

	lmics.tw OR third world.tw OR lami country.tw OR lami countries.tw OR transitional country.tw OR transitional countries.tw OR emerging economies.tw OR emerging nation.tw OR emerging nations.tw	
57	55 or 56	LMICs
58	30 and 35 and 43 and 65 and 57	

Coding of intervention and target sample characteristics

As described in the main text, each paper is coded with respect to the intervention type; the target population; the intervention components; and the intervention duration. This information is summarized in Supplementary Table S2 below.

Table S2: Study characteristics as coded for meta-regression analysis

Paper	Intervention coding	Target sample coding	Intervention components	Intervention duration - full length, in years	Intervention duration - number of hours
Abramsky et al. (2014)	Community-level mobilization	No restrictions	HIV/SRH	2.8	32
Abramsky et al. (2016)	Community-level mobilization	No restrictions	HIV/SRH	2.8	32
Chatterji et al. (2020)	Joint intervention	Cohabiting	Substance use	1.5	79
Christofides et al. (2020)	Community-level mobilization	No restrictions	HIV/SRH; Substance use	1.5	72
Clark et al. (2020)	Joint intervention	Cohabiting	Substance use	0.77	36
Doyle et al. (2018)	Couples' group-level intervention	Cohabiting; youth	HIV/SRH; Parenting	0.42	45
Dunkle et al. (2020)	Joint intervention	Cohabiting	Substance use	1.5	79
Fawzi et al. (2019)	Joint intervention	No restrictions	HIV/SRH	0.21	35
Ferrari et al. (2010)	Couples' group-level intervention	No restrictions	No additional components	Not specified	Not specified
Gibbs et al. (2020a)	Joint intervention	Youth	Substance use; HIV/SRH; Economic	0.19	63
Gibbs et al. (2020b)	Joint intervention	No restrictions	Economic	1	104
Gupta et al. (2013)	Women's group-level intervention	No restrictions	No additional components	0.31	16
Halim et al. (2019)	Men's group-level intervention in arm 1; Joint intervention in arm 2	Cohabiting	HIV/SRH; Parenting	0.5	24
Harvey et al. (2021)	Women's group-level intervention	No restrictions	No additional components	0.38	20
Hossain et al. (2014)	Men's group-level intervention	No restrictions	No additional components	0.31	16
Jewkes et al. (2008)	Joint intervention	Youth	HIV/SRH	0.15	50
Jones et al. (2014)	Joint intervention	Cohabiting	HIV/SRH	0.08	8
Kapiga et al. (2021)	Women's group-level intervention	No restrictions	No additional components	0.38	20
Maman et al. (2020)	Men's group-level intervention	No restrictions	HIV/SRH; Economic	2	Not specified

Minnis et al. (2015)	Joint intervention in arm 1 (both men's and women's groups are conducted separately); couples' group intervention in arm 2	Cohabiting; youth	HIV/SRH; Substance use	0.019	6
Naved et al. (2018)	Joint intervention in both arms	Youth	No additional components	1.67	26
Ogum et al. (2020)	Community-level mobilization	No restrictions	No additional components	1.5	Not specified
Pettifor et al. (2018)	Community-level mobilization	No restrictions	HIV/SRH; Substance use	2	Not specified
Pronyk et al. (2006)	Women's group-level intervention	No restrictions	HIV/SRH; Economic	1.17	20
Settergren et al. (2018)	Joint intervention	No restrictions	No additional components	2	Not specified
Sharma et al. (2020)	Couples' group-level intervention in arm 2; women's group-level intervention in arm 2; men's group-level intervention in arm 3	Cohabiting	HIV/SRH	0.13	38
Skar et al. (2021)	Women's group-level intervention in both arms; note in one arm, the group-level intervention is focused only on child development questions, and in the second arm, it includes a violence-related curriculum. The first arm is dropped from analysis.	No restrictions	Parenting	Not specified	12
Vaillant et al. (2020)	Men's group-level intervention	No restrictions	No additional components	0.31	48
Wagman et al. (2015)	Community-level mobilization	No restrictions	HIV/SRH	4	Not specified
Wechsberg et al. (2013)	Women's group-level intervention; in arm 1, this is focused on violence and other risk factors, and in arm 2, the groups receive information around nutrition and exercise (an "equal attention" arm). The second arm is dropped from analysis.	Youth	HIV/SRH; Substance use	Not specified	4

Methodological details: Meta-analysis

Our primary reported findings are derived from the estimation of a multilevel model that includes all available estimated effects for each trial (all outcomes of interest as specified in the systematic review protocol, as reported across multiple arms if applicable), and accounts for the dependence across estimated effects within the same trial. Accordingly, the first level captures heterogeneity across trials, and the second level captures heterogeneity across effect estimates within the same trial (corresponding to different outcome variables reported, and/or different treatment arms). Again, we conducted the multilevel meta-analysis separately for unadjusted and adjusted estimates, and report a multilevel I^2 statistic to characterize heterogeneity across estimated effects.[7] We also report the results using an extended forest plot adopted to capture the three-level model.[8]

We then estimated two meta-regressions using the multilevel model in order to analyze the effects of study characteristics on the effect size. The meta-regressions estimated can be written as follows. Both specifications were estimated separately using unadjusted and adjusted effect sizes.

$$\theta_i = \beta_0 + \beta_1 \text{ Women}_i + \beta_2 \text{ Men}_i + \beta_3 \text{ Couples}_i + \beta_4 \text{ Comm}_i + \beta_5 \text{ Youth}_i + \beta_6 \text{ Cohabiting}_i + \varepsilon_i + \zeta_i$$

$$\theta_i = \beta_0 + \beta_1 \text{ HIV/SRH}_i + \beta_2 \text{ Substance}_i + \beta_3 \text{ Economic}_i + \beta_4 \text{ Parenting}_i + \beta_5 \text{ High\_intensity}_i + \beta_6 \text{ Long\_duration}_i + \varepsilon_i + \zeta_i$$

The regression coefficients capture how the intervention effects in the subgroups characterized by different interventions, target populations, intervention components, or duration characteristics differ from the reference group. ε_i captures the sampling error (the deviation between the effect size estimated and the true effect size) and ζ_i indicates that the true effect size is sampled from a distribution of effect sizes. A statistically significant ($p<.05$) coefficient indicates that there is a linear association between the effect estimate and the specified study characteristic.

Figure S1: Effect of community- or group-based interventions on intimate partner violence (experience of IPV variables only; unadjusted study-level estimates and adjusted study-level estimates)

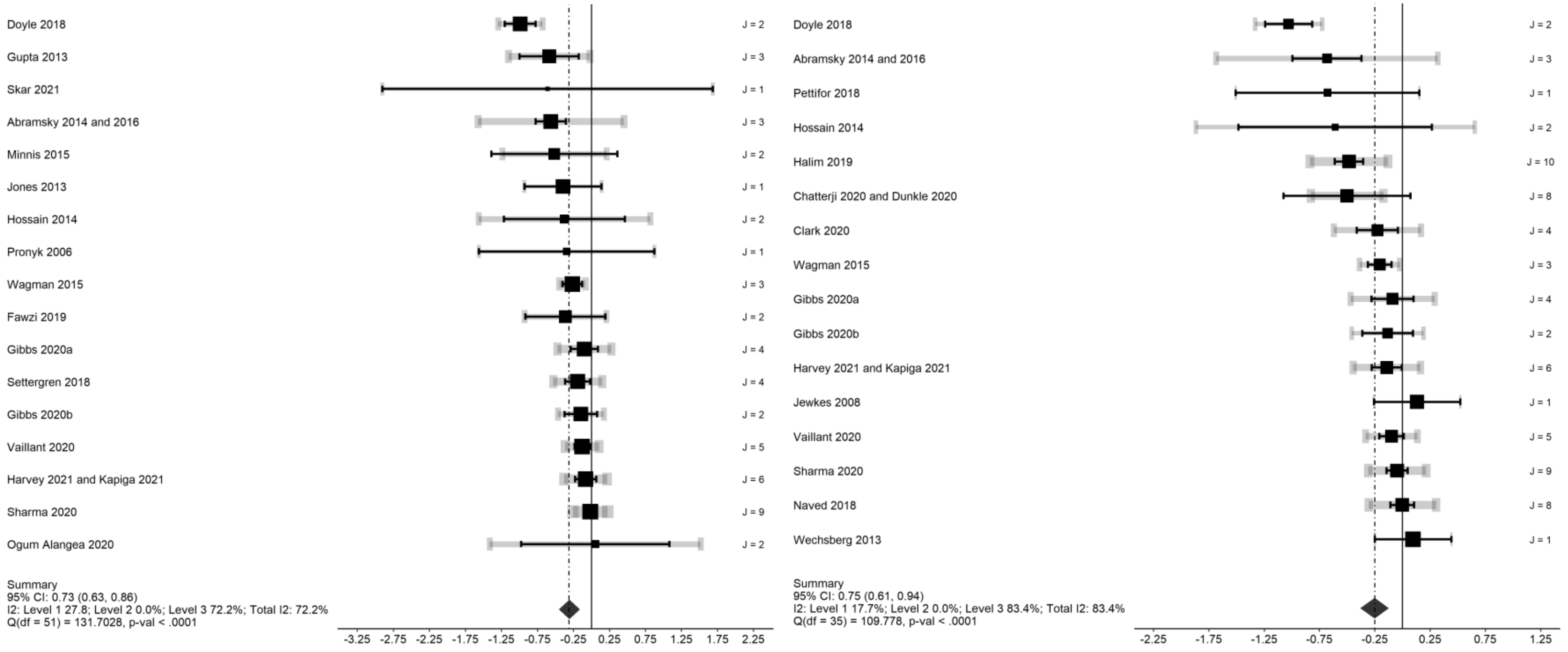

Figure S2: Effect of community- or group-based interventions on intimate partner violence (sensitivity analysis for trials including multiple papers; unadjusted study-level estimates and adjusted study-level estimates)

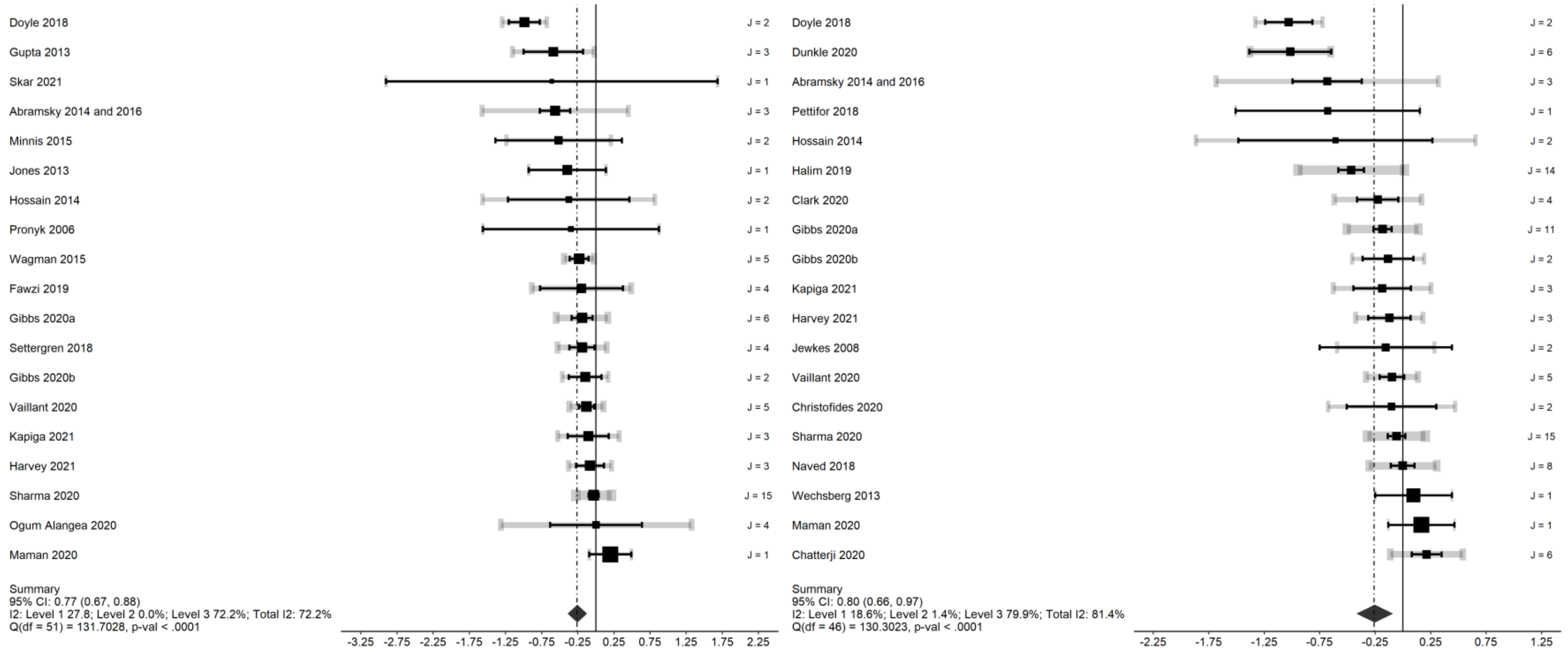

Figure S3: Effect of community- or group-based interventions on intimate partner violence (primary outcome only; unadjusted study-level estimates and adjusted study-level estimates)

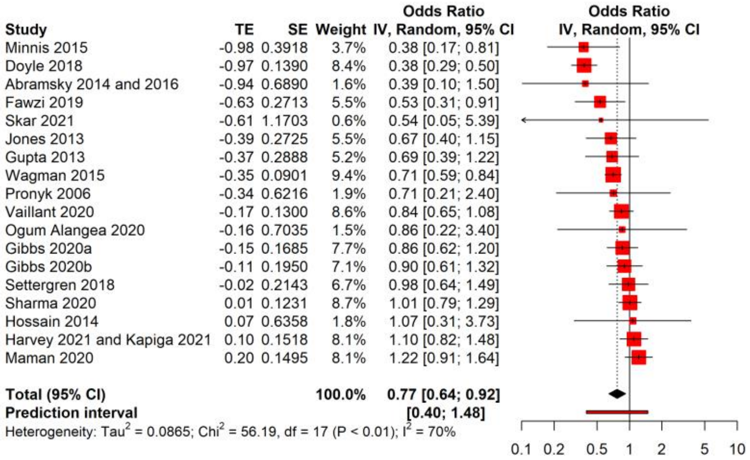

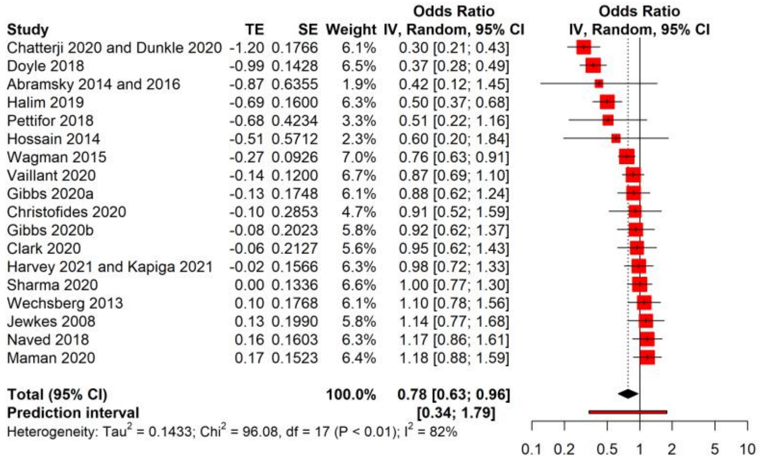

Figure S4: Effect of community- or group-based interventions on intimate partner violence (sensitivity analysis excluding trials characterized by ROB in two or more domains; unadjusted study-level estimates and adjusted study-level estimates)

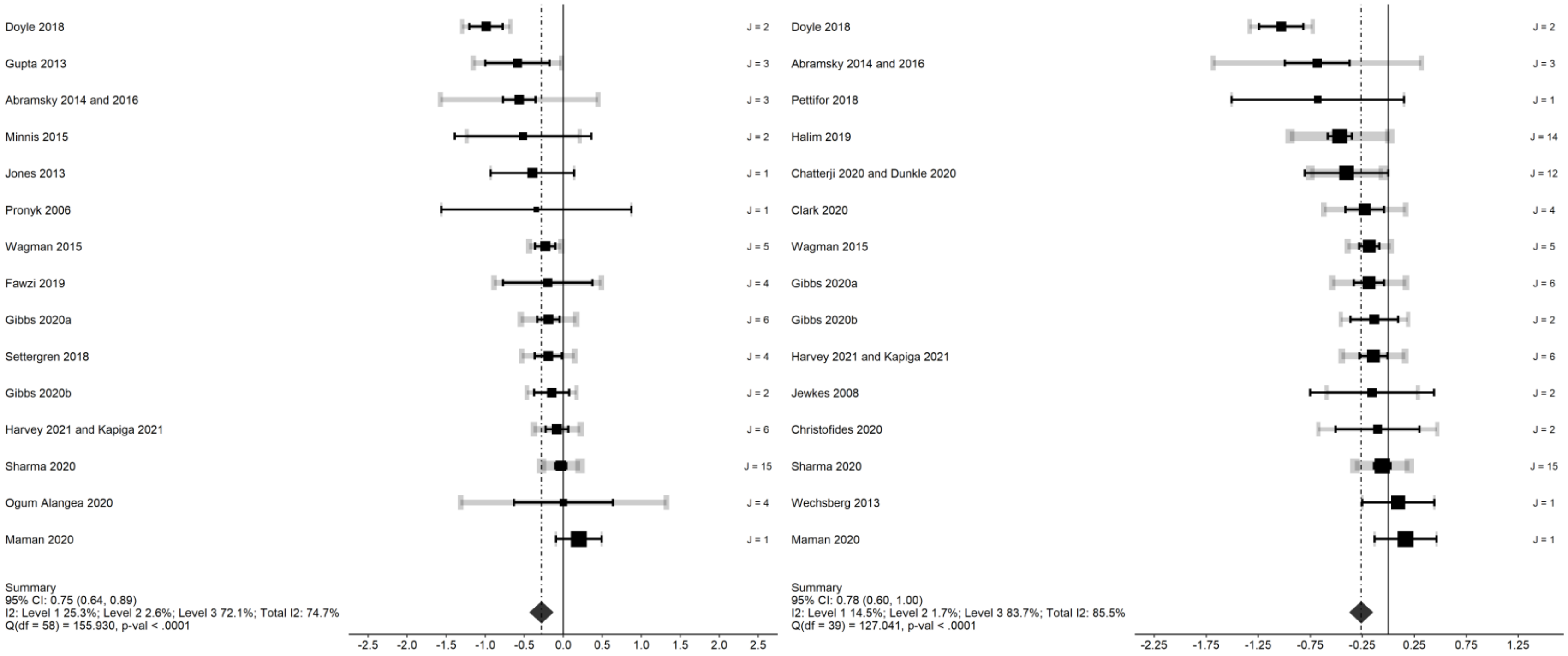

Figure S5: Effect of community- or group-based interventions on women's past-year experience of physical intimate partner violence (unadjusted study-level estimates and adjusted study-level estimates)

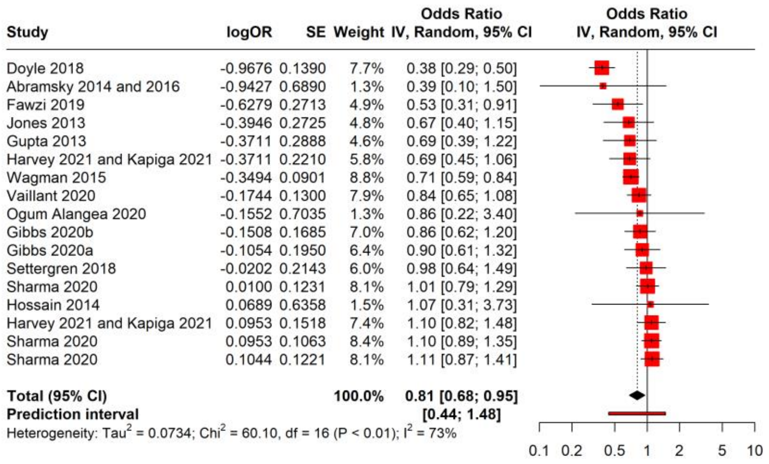

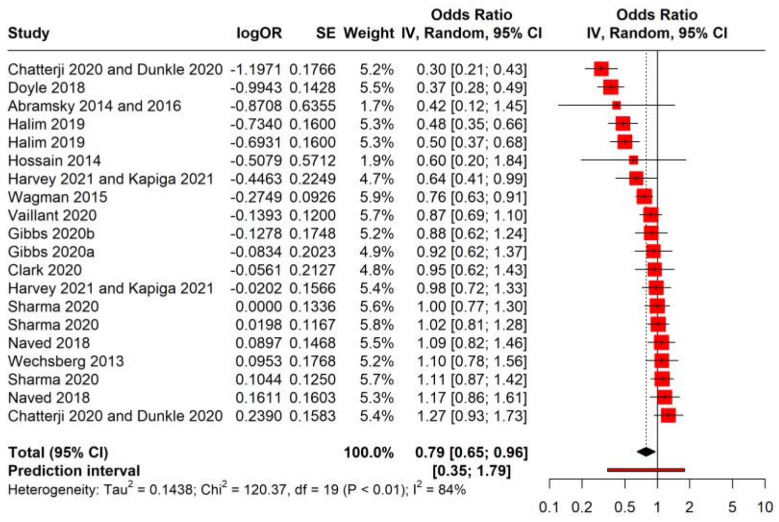

Figure S6: Effect of community- or group-based interventions on women's past-year experience of sexual intimate partner violence (unadjusted study-level estimates and adjusted study-level estimates)

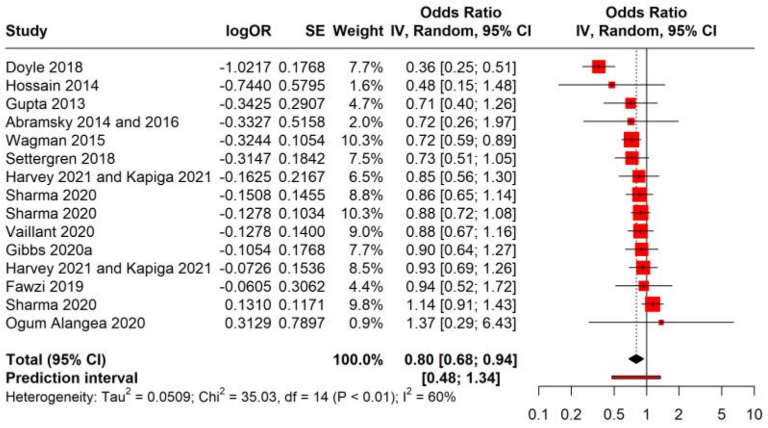

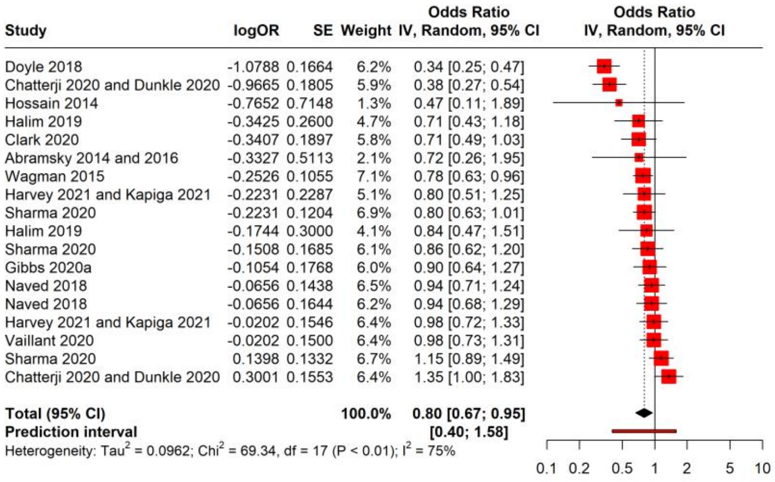

Figure S7: Effect of community- or group-based interventions on women's past-year experience of emotional intimate partner violence (unadjusted study-level estimates and adjusted study-level estimates)

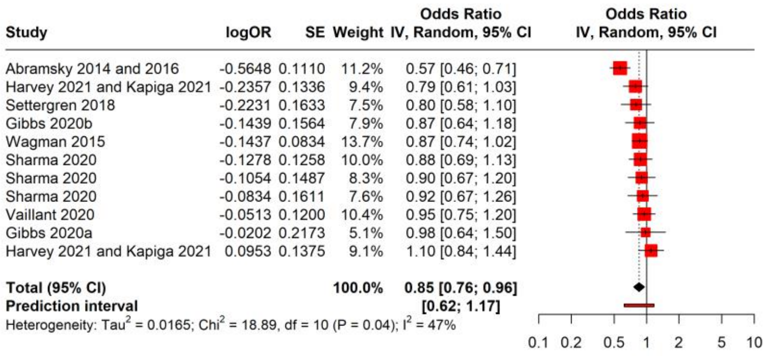

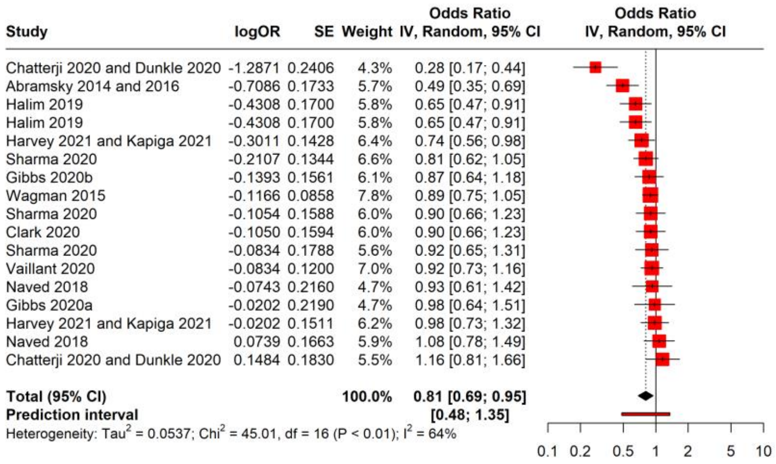

Figure S8: Effect of community- or group-based interventions on women’s past-year experience of economic intimate partner violence (unadjusted study-level estimates and adjusted study-level estimates)

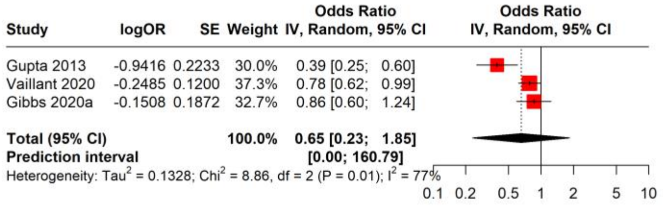

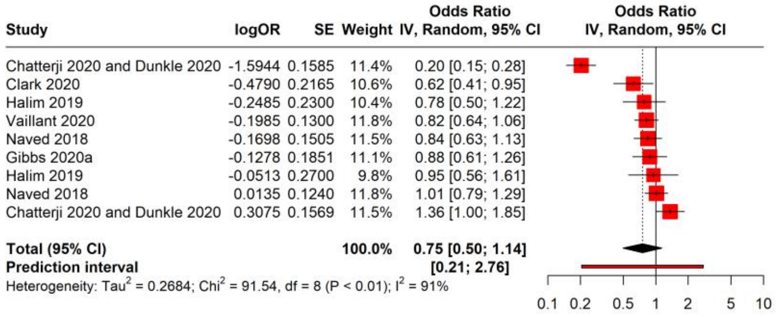

Figure S9: Effect of community- or group-based interventions on men's past-year perpetration of physical intimate partner violence (unadjusted study-level estimates and adjusted study-level estimates)

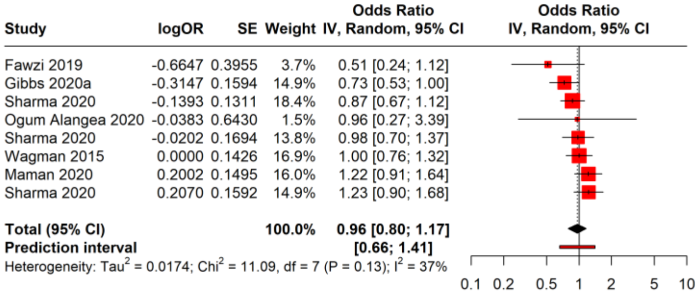

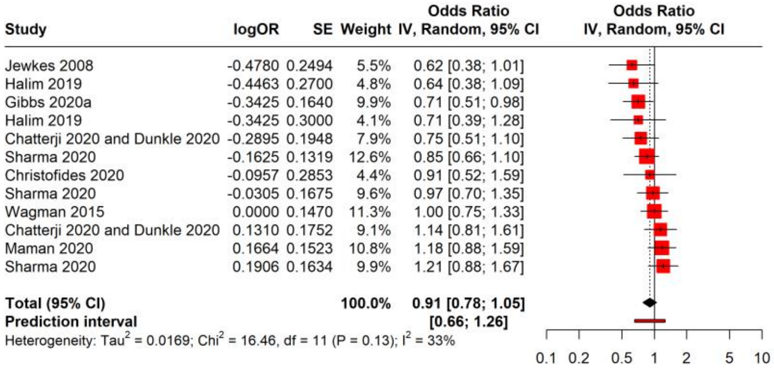

Figure S10: Effect of community- or group-based interventions on men's past-year perpetration of sexual intimate partner violence (unadjusted study-level estimates and adjusted study-level estimates)

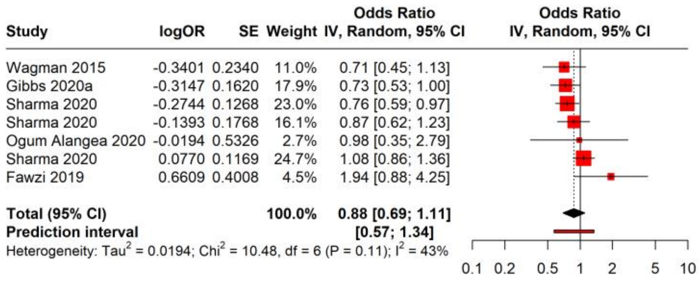

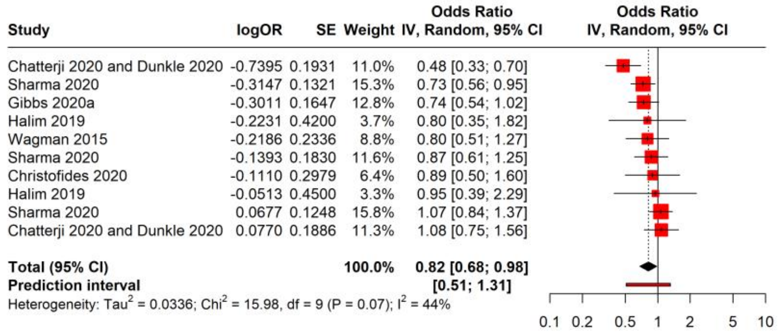

References

1. Yakubovich AR, Stöckl H, Murray J, et al. Risk and protective factors for intimate partner violence against women: systematic review and meta-analyses of prospective-longitudinal studies. *Am J Public Health*. 2018;108(7):e1-e11. doi:10.2105/AJPH.2018.304428

2. Sterne JAC, Savović J, Page MJ, et al. RoB 2: a revised tool for assessing risk of bias in randomised trials. *bmj*. 2019;366.

3. Eggers Del Campo I, Steinert JI. The effect of female economic empowerment interventions on the risk of intimate partner violence: a systematic review and meta-analysis. *Trauma Violence Abuse*. 2022;22(3):810-826. doi:10.1177/1524838020976088

4. Turner DT, Riedel E, Kobeissi LH, et al. Psychosocial interventions for intimate partner violence in low and middle income countries: A meta-analysis of randomised controlled trials. *J Glob Health*. 2020;10(1):10409. doi:10.7189/jogh.10.010409

5. Cork C, White R, Noel P, Bergin N. Randomized controlled trials of interventions addressing intimate partner violence in sub-Saharan Africa: a systematic review. *Trauma Violence Abuse*. 2020;21(4):643-659. doi:10.1177/1524838018784585

6. Awolaran O, Olubumuyi O, OlaOlorun F, Assink M, van Rooij F, Leijten P. Interventions to reduce intimate partner violence against women in low- and middle-income countries: A meta-analysis. *Aggress Violent Behav*. 2022;64:101746. doi:https://doi.org/10.1016/j.avb.2022.101746

7. Cheung MWL. Modeling dependent effect sizes with three-level meta-analyses: a structural equation modeling approach. *Psychol Methods*. 2014;19(2):211.

8. Fernández-Castilla B, Declercq L, Jamshidi L, Beretvas N, Onghena P, Van den Noortgate W. Visual representations of meta-analyses of multiple outcomes: extensions to forest plots, funnel plots, and caterpillar plots. *Methodology*. 2020;16(4 SE-Original Article):299-315. doi:10.5964/meth.4013
